# Does Mycorrhizal Biotechnology Modulate Lectin Accumulation in the Stem of *Schinus terebinthifolia* Raddi Seedlings?

**DOI:** 10.1021/acsomega.5c07898

**Published:** 2025-09-12

**Authors:** Caio Bezerra Barreto, Francisco Chagas Barbalho Neto, Carmelo José Albanez Bastos-Filho, Qiang-Sheng Wu, Michele Dalvina Correia da Silva, Fábio Sérgio Barbosa da Silva

**Affiliations:** 1 Laboratório de Análises, Pesquisas e Estudos em Micorrizas (LAPEM), Programa de Pós-graduação em Biologia Celular e Molecular Aplicada, 498944 Universidade de Pernambuco (UPE), Rua Arnóbio Marques, 310, Santo Amaro , Recife, PE 50100-130, Brazil; 2 89113 Universidade do Estado do Rio Grande do Norte (UERN), Rua Almino Afonso, 478, Centro , Mossoró, RN 59610-210, Brazil; 3 Programa de Pós-graduação em Engenharia de Sistemas, Escola Politécnica de Pernambuco, Universidade de Pernambuco (UPE), Rua Benfica, 455, Madalena , Recife, PE 50720-001, Brazil; 4 Hubei Key Laboratory of Spices and Horticultural Plant Germplasm Innovation and Utilization, College of Horticulture and Gardening, Yangtze University, Jingzhou 434025, China; 5 Universidade Federal Rural do Semi-Árido (UFERSA), Av. Francisco Mota, 572, Bairro Costa e Silva, Mossoró, RN 59625-900, Brazil; 6 117110 Universidade de Pernambuco, Av. Agamenon Magalhães, S/N, Santo Amaro, Recife, PE 50100-010, Brazil

## Abstract

It is well-established that mycorrhizal symbiosis can alter lectin expression in plant roots, whereas little is known about its role in lectin accumulation in other plant organs and whether such behavior is related to the production of antioxidant secondary metabolites. This study aimed to evaluate whether the lectin accumulation profile in the stems of *Schinus terebinthifolia* Raddi seedlings is modulated in response to inoculation with an arbuscular mycorrhizal fungus (AMF) consortium. A greenhouse experiment was set up with two inoculation treatments: a noninoculated control and an AMF treatment (consortium of *Acaulospora longula*, *Entrophospora etunicata*, and *Dentiscutata heterogama*). After 191 days, stem tissues were harvested to prepare aqueous extracts. Primary and secondary metabolites were quantified spectrophotometrically, and *in vitro* antioxidant activity was evaluated. The hemagglutinating activity assay was performed to detect lectins, and the specific hemagglutinating activity (SHA) was determined. The AMF consortium significantly (*p* ≤ 0.01) enhanced the accumulation of metabolites, antioxidant activity, and SHA by over 110%, in comparison to control plants. The anabolism of carbohydrates, proteins, and phenols was highly correlated (*r* ≥ 0.8) with stem SHA. To our knowledge, this is the first study demonstrating the effect of mycorrhizal symbiosis on the specific hemagglutinating activity of plant extracts, revealing the presence of bioactive lectins in *S. terebinthifolia* stems and its relation to the production of other bioactive compounds. It suggests that AMF can quantitatively and qualitatively modulate lectin accumulation, a process closely tied to the host’s anabolism.

## Graphical abstract

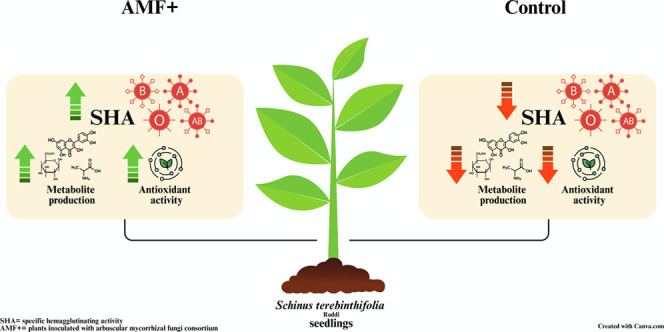

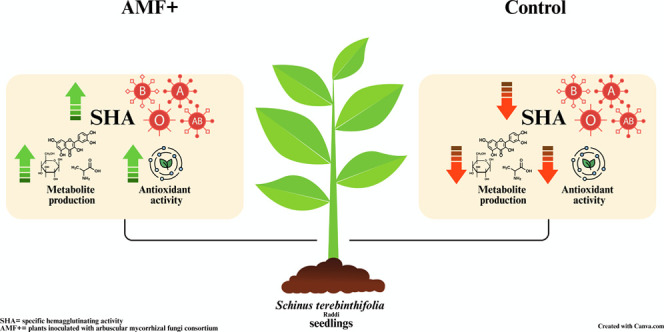

## Introduction

The mechanisms that explain the effects of mycorrhizal biostimulants in biochemistry and phytochemistry of plant hosts have been widely studied,^1–3^ with particular attention to metabolic profile changes mediated by arbuscular mycorrhizal fungi (AMF). In this context, favoring the activity of key secondary metabolism enzymes,^4^ greater accumulation of carbohydrates,^5^ and the production of signaling molecules^6^ are some of the factors that may explain the outstanding accumulation of metabolites in mycorrhizal plants.

Primary metabolite production is intimately related to mycorrhizal symbiosis, considering the supply of carbohydrates in the host–AMF relationship.^7^ In addition to that, formation of mycorrhizae can modulate the accumulation of total proteins,^8^ or of specific molecules, such as proline, with the production of the latter being one of the proposed mechanisms for a greater tolerance to abiotic stress mediated by AMF.^9^ Among these proteins, lectins, a class of carbohydrate-binding proteins,^10^ play critical roles in plant–microbe interactions, including the establishment of mycorrhizal symbiosis,^11^ as well as performing several physiological roles in other plant organs.^12,13^ Although the expression of specific lectins, induced in mycorrhizal roots, has been reported,^14,15^ little is known about the role of mycorrhizal symbiosis in modulating the activity of lectins in aerial tissues, such as stems, and its potential relationship in enhancing the production of other metabolites.

To address this knowledge gap, a method for evaluating the presence of lectins in plant tissues is the use of the hemagglutination activity assay.^16^ In this assay, lectins can interact with carbohydrates present on the surface of erythrocytes, forming a network of connections between cells and promoting their agglutination.^17,18^ Considering the relation between the hemagglutinating activity and the protein concentration of a sample, it is possible to determine the specific hemagglutinating activity, which is indicative of lectin accumulation.^19^

Therefore, we selected *Schinus terebinthifolia* Raddi, a highly mycotrophic plant^20^ with cosmetic and medicinal significance, as the host, to test two hypotheses: (1) the inoculation with an AMF consortium modulates lectin accumulation in stems; (2) the hemagglutinating activity is correlated with the production of stem secondary metabolites and antioxidant activity in mycorrhizal *S. terebinthifolia* seedlings.

## Materials and Methods

To evaluate whether the inoculation of an AMF consortium can modulate the production of lectins in the stem of *S. terebinthifolia*, a greenhouse experiment was carried out using a completely randomized design with two inoculation treatments: noninoculated control and AMF+ (a consortium of*Acaulospora longula* Spain & N.C. Schenck, *Entrophospora etunicata* (W.N. Becker & Gerd.) Błaszk., Niezgoda, B.T. Goto & Magurno, and *Dentiscutata heterogama* (T.H. Nicolson & Gerd.) Sieverd., F.A. Souza & Oehl), in five replicates per treatment. These species were chosen because the inoculation of consortia containing three AMF isolates improves the mycorrhizal benefits to growth^21^ and secondary compound anabolism^22^ in *S. terebinthifolia*seedlings. The inocula of the tested AMF were produced using *Panicum miliaceum* L. as the host, according to Silva et al.^23^ The soil inoculum was kept refrigerated (4 °C) until use.^24^ The mean infection percentage (MIP)^25^ of each isolate was 53% for *A. longula*, 6% for *D. heterogama*, and 37% for *E. etunicata*. The AMF isolates are registered on *Sistema Nacional de Gestão do Patrimônio Genético e do Conhecimento Tradicional Associado* (*SisGen*) under the registration ABC3019.

Plantlets of *S. terebinthifolia* were obtained from seeds (Germiverde Comércio de Sementes Ltd.a., Buritama, SP, Brazil), which were disinfested with NaClO (0.05%) and germinated in vermiculite (Urimamã Mineração Ltd.a., Santa Maria da Boa Vista, PE, Brazil) that was previously sterilized (121 °C for 30 min, in two consecutive days). When plantlets had two definitive leaves, they were transferred into black polyethylene bags (8 cm × 12 cm × 32 cm) containing a mixture of sand (Granumix, Vitória de Santo Antão, PE, Brazil), vermiculite (Urimamã Mineração Ltd.a., Santa Maria da Boa Vista, PE, Brazil), and vermicompost (All Garden, Jaguariúna, SP, Brazil) (4.5;4.5;1, v/v). In that moment, according to the inoculation treatment, soil inoculum was added or not (control), containing 300 glomerospores (approximately 100 glomerospores of *A. longula*, 100 glomerospores of *E. etunicata*, and 100 glomerospores of *D. heterogama*). The noninoculated control received a filtrate of soil inoculum free of glomerospores.^26^

Plants were kept in the greenhouse in environmental conditions of temperature (*T*_min_ 23.1 °C; *T*_max_ 34.8 °C) and relative air humidity (RH_min_ 56%; RH_max_ 97%) for 191 days until reaching at least 25 cm of height and 3.0 mm of stem diameter.^27^ During this period, the plants were watered at 8 am every other day, to maintain 70% of the total substrate pore volume filled with water. Similarly, during the third and fifth months, the cultivation substrate was watered every other day with a modified Hoagland solution without Tris–HCl buffer and once a week with distilled water, to avoid salt accumulation.^23^ At the end of the experiment, the aerial part was harvested, and the stems and leaves of the seedlings were separated. Only the stems were used for the evaluations.

Aqueous extracts from the stems were prepared to evaluate the production of primary metabolites (total soluble carbohydrates and total proteins), secondary compounds (total phenols, flavonols, and saponins), antioxidant (phosphomolybdenum complex reduction, expressed in vitamins C and E), and hemagglutinating activities. The analyses were performed in technical quintuplicate. After oven-drying (45 °C) (Quimis, Diadema, SP, Brazil), the stem was sectioned. Aliquots of 50 mg were added with 10 mL of distilled water and subjected to ultrasonic-assisted extraction (40 kHz, 100W at 20 °C)^28^ (Solidsteel, Piracicaba, SP, Brazil) for 30 min. The extracts were filtered in qualitative paper filters (Unifil, Carvalhaes Produtos para Laboratório Ltd.a., Alvorada, RS, Brazil) and stored at −18 °C.

Total soluble carbohydrates were dosed by the phenol-sulfuric method^29^ using a standard curve of glucose (*y* = 0.0003*x* – 0.0189; *R* ^2^= 0.9962) (Cromato Produtos Químicos Ltd.a., Diadema, SP, Brazil). Total proteins were quantified according to the methodology of Bradford,^30^ with a standard curve of bovine serum albumin (*y* = 0.0007*x* – 0.0063; *R* ^*2*^ = 0.9997) (Sigma-Aldrich, San Luis, Missouri, United States).

To spectrophotometrically quantify secondary metabolites, total phenolics were dosed by the Folin-Ciocalteu method,^31^ using a standard curve of gallic acid (*y* = 0,0071*x* + 0.0352; *R* ^*2*^ = 0,9976) (Vetec Ltd.a., Duque de Caxias, RJ, Brazil), total flavonols, by the aluminum chloride complexation method,^32^ expressed in quercetin equivalents (*y* = 0.0063*x* – 0.0231; *R* ^*2*^ = 0.9982) (Sigma-Aldrich, San Luis, MO, United States), and total saponins, by the cobalt chloride complexation method,^32^ using a standard curve of saponin (*y* = 0.0009*x* + 0,0045; *R* ^*2*^ = 0.9926) (INLAB Confiança, São Paulo, SP, Brazil).

The *in vitro* antioxidant activity was evaluated by phosphomolybdenum complex reduction,^31^ with the results expressed as percentage, in relation to ascorbic acid (Dinâmica Química Contemporânea Ltd.a., Indaiatuba, SP, Brazil) and to tocopherol (Sigma-Aldrich, San Luis, Missouri, United States), both at the concentration of 0.25 g L^–1^.

To evaluate the presence of lectin activity in the extracts, hemagglutinating activity (HA) assays were conducted.^33^ Microplates with 96 V-shaped well bottoms (Kasvi, Pinhais, PR, Brazil) had 50 μL of saline solution (NaCl 0.15M, in H_2_O, Vetec Ltd.a., Duque de Caxias, RJ, Brazil) added. In sequence, 50 μL of the stem extracts was added to the second well in each assay, and serial dilutions were made. Lastly, 50 μL of a suspension of glutaraldehyde-fixed erythrocytes (2.5%, v/v in NaCl 0.15M)^34^ was added to all the wells, and the samples were left for 45 min at 20 °C. Following that, the HA titer was determined as the highest dilution that presented total hemagglutination^19^ (Figure S1). For the hemagglutinating activity assays, human erythrocytes from groups A, AB, B, and O were used. The research was conducted in conformity with the Declaration of Helsinki. No information identifies the participants who volunteered to donate blood samples to the research. The Research Ethics Committee *Comitê de Ética em Pesquisa em Seres Humanos da Universidade do Estado do Rio Grande do Norte* (CEP-UERN) (approval no. 7.597.854) approved the approaches carried out in this study through the Certificate of Presentation of Ethical Appreciation (CAAE: 88068525.6.0000.5294).

Additionally, to microscopically evaluate the hemagglutination pattern of the samples, at the end of the hemagglutinating activity assay, aliquots of 10 μL from the well containing the smallest dilution that presented HA were placed in slides, covered with coverslips, and observed in an inverted light microscope (TCM 400, Labomed, Fremont, California, United States of America). Scale bars were added with ImageJ.^35^ The hemagglutination pattern caused by the extracts is shown in Figure S2.

The specific hemagglutinating activity (SHA) was determined with the following equation: SHA = HA titer/total proteins (mg mL^–1^).^19^ A scheme of the methodology used in this experiment is presented in Figure S3.

The obtained data had their means compared with the *t*-test (1%). Using the Google collaborative platform in Python language, agglomerative hierarchical clustering algorithm and K-means algorithm analyses were conducted. The correlation between the variables was evaluated by Spearman’s correlation (ρ).

## Results

The inoculation with AMF consortium positively modulated the lectin activity in the stem of *S. terebinthifolia* seedlings (Figure 1).

**Figure 1:**
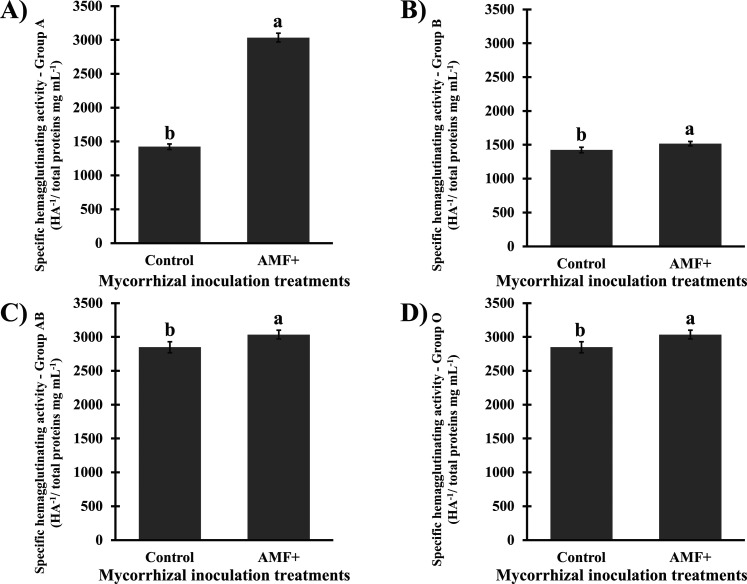
Specific hemagglutinating activity (SHA) (hemagglutinating activity titer, HA^–1^/total proteins mg mL^–1^) of aqueous stem extracts from *Schinus terebinthifolia* Raddi seedlings noninoculated (control) or inoculated with arbuscular mycorrhizal fungi (AMF+). SHA of the extracts on group A human erythrocytes (A), CV = 2.42%, on group B (B), CV = 2.50%, on group AB, CV = 2.50%, and on group O, CV = 2.50%. Means (*n* = 5; bars = standard deviation) followed by the same letter do not differ according to the *t*-test (*p* ≤ 0.01). CV = coefficient of variation. HA^–1^ = hemagglutinating activity titer.

Stems of plants associated with AMF had increases of up to 113% in specific hemagglutinating activity, in comparison with noninoculated plants (Figure 1). The highest difference between mycorrhizal seedlings and those noninoculated was reported for SHA with group A erythrocytes (Figure 1A). The SHA was highly correlated (*r* ≥ 0.8) with the production of total proteins, total soluble carbohydrates, and total phenols (Figure 2), suggesting a coordinated modulation of primary and secondary metabolism in response to AMF symbiosis.

**Figure 2:**
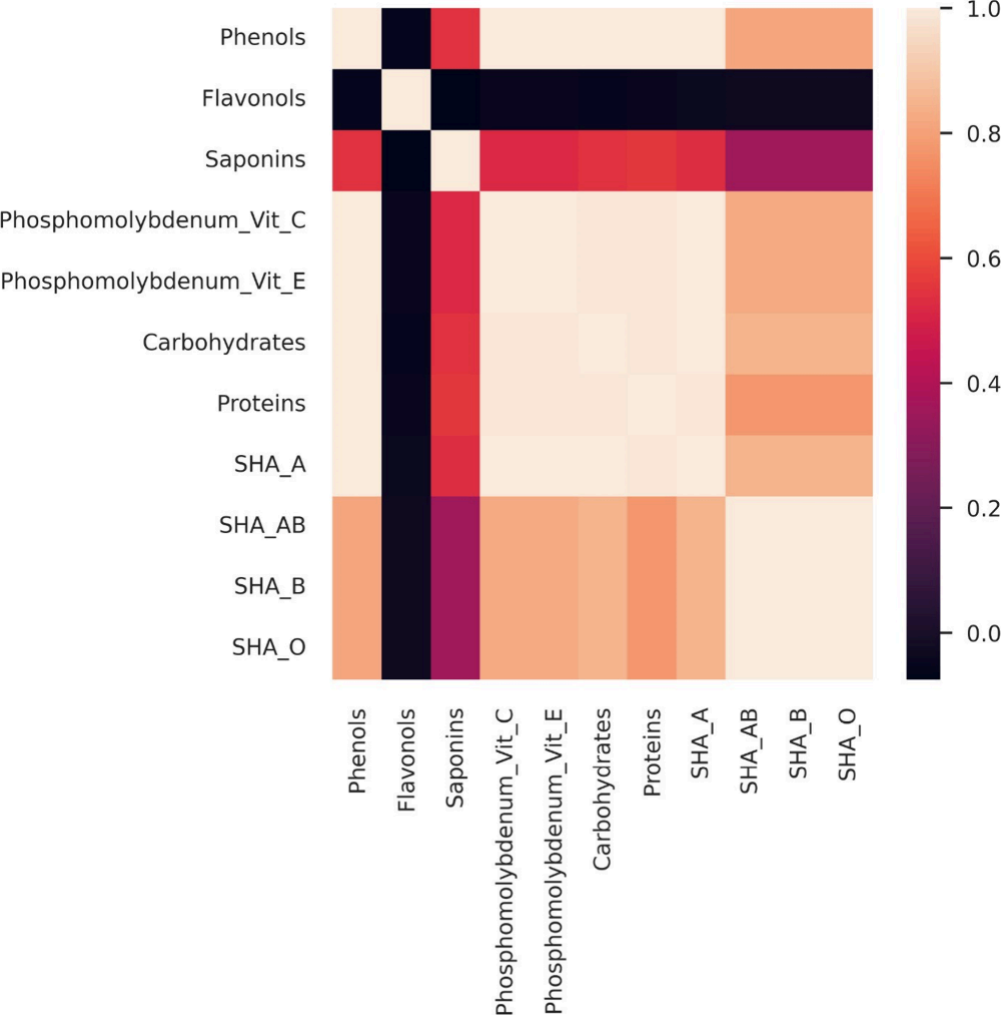
Correlation map of the variables evaluated in aqueous stem extracts of *Schinus terebinthifolia* Raddi seedlings noninoculated (control) or inoculated with arbuscular mycorrhizal fungi (AMF+). SHA A, SHA AB, SHA B, and SHA O = specific hemagglutinating activity of the extracts on human erythrocytes from groups A, AB, B, and O, respectively. Phosphomolybdenum Vit C and Vit E = *in vitro* antioxidant activity of phosphomolybdenum complex reduction with results expressed as percentage, in relation to vitamin C and vitamin E, respectively.

Furthermore, association with the AMF consortium enhanced the biosynthesis of total soluble carbohydrates and total proteins in stems of *S. terebinthifolia* seedlings (Figure 3). Stems of seedlings associated with AMF accumulated about 87% more proteins (3.44 mg g^–1^ dry matter) in comparison with noninoculated plants (1.84 mg g^–1^ dry matter). Similarly, production of soluble carbohydrates was enhanced by over 80% in the stems of AMF+ plants (8.86 mg g^–1^ dry matter), in comparison to those of seedlings not associated with the mycorrhizal consortium (4.86 mg g^–1^ dry matter).

**Figure 3:**
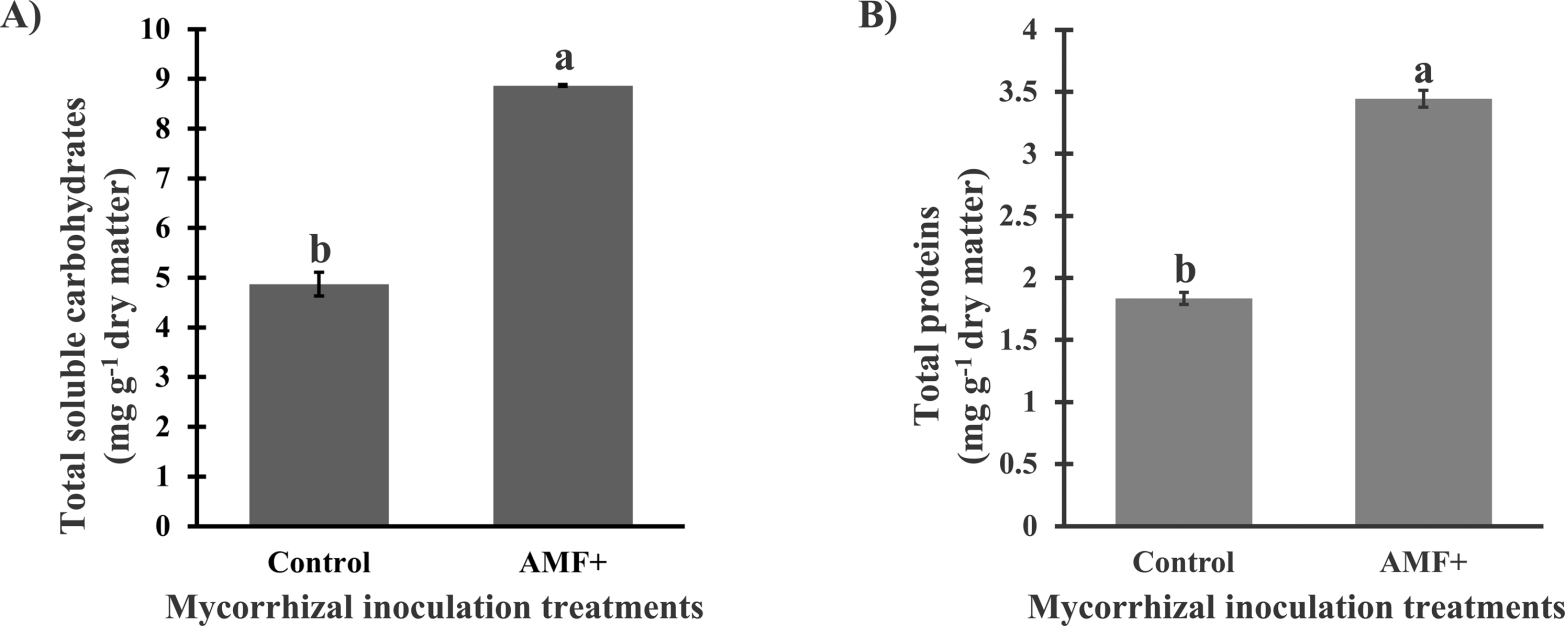
Concentration (mg g^–1^ dry matter) of primary metabolites in aqueous stem extracts from *Schinus terebinthifolia* Raddi seedlings noninoculated (control) or inoculated with arbuscular mycorrhizal fungi (AMF+). (A) total soluble carbohydrates, CV = 2.46%; (B) total proteins, CV = 2.46%. Means (*n* = 5; bars = standard deviation) followed by the same letter do not differ according to the *t*-test (*p* ≤ 0.01). CV = coefficient of variation.

The production of stem primary metabolites was highly correlated (*r* ≥ 0.8) with the production of total phenols (Figure 2). The association with the AMF consortium also favored the accumulation of total phenols (682 mg g^–1^ dry matter) and total saponins (0.63 mg g^–1^ dry matter) in stems of *S. terebinthifolia* seedlings, in comparison to noninoculated seedlings (Figure 4). On the other hand, plants associated or not with AMF did not differ in the accumulation of total flavonols (Figure 4).

**Figure 4:**
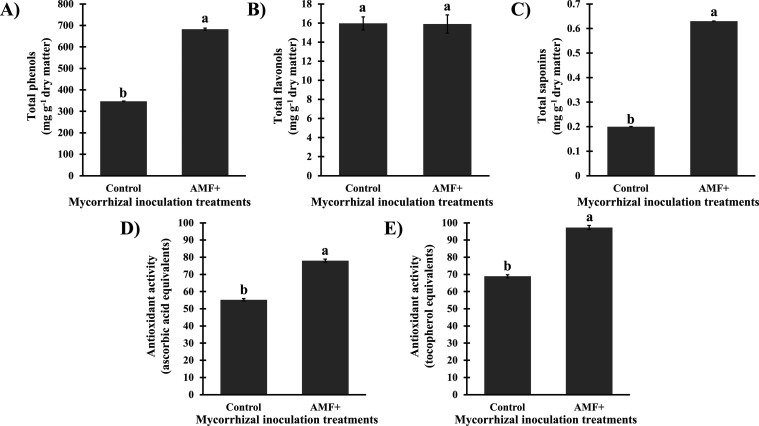
Concentration (mg g^–1^ dry matter) of secondary metabolites and antioxidant activity in aqueous stem extracts from *Schinus terebinthifolia* Raddi seedlings noninoculated (control) or inoculated with arbuscular mycorrhizal fungi (AMF+). (A) total phenols, CV = 0.72%; (B) total flavonols, CV = 5.21%; (C) total saponins, CV = 0.72%; (D) antioxidant activity of phosphomolybdenum complex reduction, in comparison with ascorbic acid (0.25 g L^–1^), CV = 1.29%; (E) antioxidant activity of phosphomolybdenum complex reduction, in comparison with tocopherol (0.25 g L^–1^), CV = 1.29%. Means (*n* = 5; bars = standard deviation) followed by the same letter do not differ according to the *t*-test (*p* ≤ 0.01). CV = coefficient of variation.

Likewise, the antioxidant activity was also enhanced in the stems of *S. terebinthifolia* seedlings associated with the AMF consortium (Figure 4). The accumulation of total phenols, total soluble carbohydrates, and total proteins was highly correlated (*r* ≥ 0.8) with the antioxidant activity of the extracts (Figure 2). The inoculation of the consortium of*A. longula*, *E. etunicata*, and *D. heterogama* enhanced the *in vitro* antioxidant activity of the stem extracts by over 40%, in comparison with noninoculated plants.

Hierarchical clustering analysis revealed clear separation between two treatments (Figure 5), with mycorrhizal and nonmycorrhizal plants forming two distinct groups.

**Figure 5:**
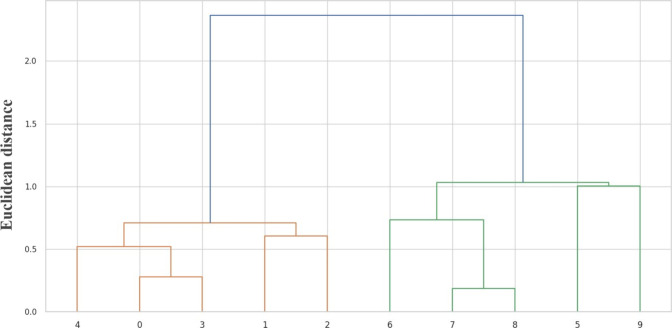
Dendrogram considering specific hemagglutinating activity, the production of primary and secondary metabolites, and the antioxidant activity in aqueous stem extracts of *Schinus terebinthifolia*Raddi seedlings noninoculated 0–4 (control) or inoculated 5–9 (AMF+) with arbuscular mycorrhizal fungi.

The production of total phenols, the *in vitro* antioxidant activity, the production of primary metabolites, and the specific hemagglutinating activity with group A erythrocytes were the variables that governed the differentiation of noninoculated plants from those associated with the AMF consortium (Figure 6).

**Figure 6:**
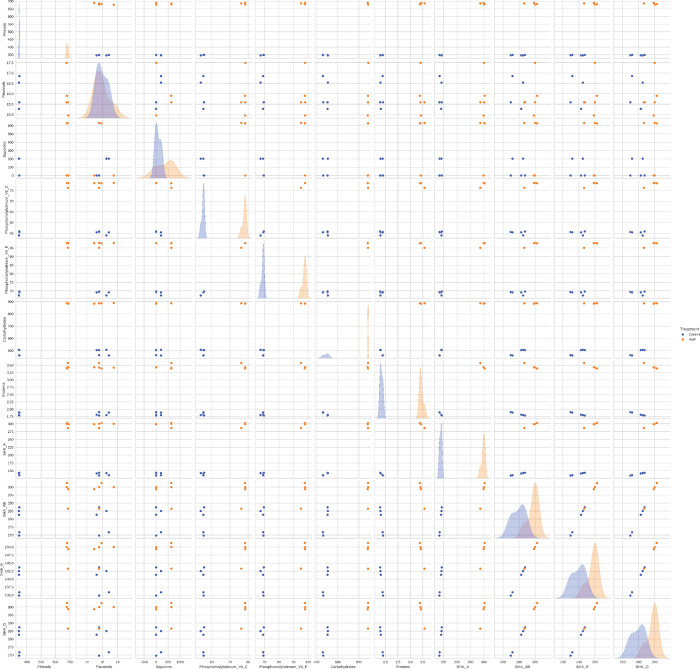
Data distribution (*K*-means = 2) considering specific hemagglutinating activity (SHA), the production of primary and secondary metabolites, and the antioxidant activity in aqueous stem extracts of *Schinus terebinthifolia* Raddi seedlings noninoculated (control) or inoculated (AMF+) with arbuscular mycorrhizal fungi. SHA A, SHA AB, SHA B, and SHA O = specific hemagglutinating activity of the extracts on human erythrocytes from groups A, AB, B, and O, respectively. Phosphomolybdenum Vit C and Vit E = *in vitro* antioxidant activity of phosphomolybdenum complex reduction, with results expressed in percentage, in relation to vitamin C and vitamin E, respectively.

## Discussion

This study provides the first evidence that mycorrhizal symbiosis modulates lectin accumulation in plant stems (Figure 1). The elevated hemagglutinating activity titers in this research (Figure 1A) strongly suggest lectin accumulation, as the hemagglutinating assay is well-established for detecting bioactive lectins.^36^ While other metabolites, such as some polyphenols, oligosaccharides, and polysaccharides, are reported as promoters of hemagglutination,^37,38^ the verified variations, for each plant group (Control and AMF+), regarding their hemagglutinating activity titers (Figure 1A), and the specific hemagglutinating activity values (Figure 1), depending on the blood group, suggests specificity in the interactions between soluble biomolecules and the surface of the erythrocytes, which also occurs between the binding sites for specific carbohydrates of lectins and their molecular targets^39^ and, in that case, the differentially expressed plasma membrane glycoconjugates in erythrocytes of groups, A, B, AB, and O. Thus, it is possible that the variable profiles of hemagglutinating activity and specific hemagglutinating activity are related to the accumulation of bioactive lectins; we also consider the possible synergism of carbohydrates, phenolics, and lectins in promoting the hemagglutinating effect. Higher concentrations of certain classes of metabolites are highly correlated with lectin accumulation (Figure 2).

Regarding the stem of the evaluated seedlings, the association with the consortium of *A. longula*, *E. etunicata*, and *D. heterogama* enhanced the anabolism of some primary and secondary metabolites and the associated antioxidant potential (Figures 3 and 4). In this context, increased accumulation of carbohydrates is one of the classical mechanisms by which mycorrhization modulates the secondary metabolism.^40^ One of the explanations is that, probably, carbohydrates and intermediates of their metabolic routes, such as phosphoenolpyruvate and erythrose-4-phosphate, have been used as precursors in the biosynthesis of phenolic compounds.^41^

One of the proposed functions for plant lectins present in the stem is controlling the transport of macromolecules in the phloem, possibly modulating plant anabolism.^42,43^ Thus, we hypothesize that the higher accumulation of bioactive lectins in the stem of *S. terebinthifolia* seedlings mediated by mycorrhizal symbiosis (Figure 1) favored the offer of photoassimilates (Figure 3), which were directed to the secondary metabolism (Figure 4). The accumulation of carbohydrates in the stem was highly correlated with the SHA, especially regarding group A erythrocytes, in the present study (Figures 2 and 3). Plants associated with AMF likely produce more lectins in specific tissues in response to higher carbohydrate accumulation, a phenomenon not yet reported in the literature.

Unsurprisingly, a high correlation was verified between the accumulation of phenols, carbohydrates, proteins, and the antioxidant activity (Figure 2); such molecules are widely known for their antioxidant potential,^44–47^ something related to the presence of amine and hydroxyl groups.^45,47,48^ It is possible that the higher accumulation of these biomolecules has contributed synergistically to the enhanced antioxidant metabolism (Figure 4).

Regarding the data distribution by K-means, in approaches that reported about the phytochemistry of mycorrhizal plants, the role of the production of total phenols and antioxidant activity was reinforced in separating or not the groups of mycorrhizal and nonmycorrhizal plants (Figure 6).^49,50^ In the present study, it is reported for the first time the importance of evaluating the presence and profile of bioactivity of hemagglutinating molecules for the differential grouping of plants inoculated or not with an AMF consortium (Figure 6).

The highest increase in SHA was verified utilizing group A erythrocytes (Figure 1). The presence of antigens with distinct carbohydrate residues in the surface of ABO group erythrocytes^51^ can explain the observed differences. In this perspective, the accumulation of lectins in inoculated plants, with higher affinity for *N*-acetyl-galactosamine, the terminal carbohydrate present in the antigen of group A,^51^ may explain the obtained results. In that context, a lectin with affinity for a *N*-acetylated amino sugar (*N*-acetyl-glucosamine) was previously isolated from *S. terebinthifolia* leaves.^52^ Therefore, we suggest that the association with AMF can qualitatively modulate the lectin profile in the host’s stem.

Conducting hemagglutinating activity inhibition assays is a way to evaluate the specificity characteristics of plant lectins.^53^ Future studies should consider the hemagglutinating activity of the extract of mycorrhizal plants in inhibition assays in relation to differing types of carbohydrates, as a way to confirm the modulation of the presence of lectins with affinity for distinct targets.

The recognized role of mycorrhizal symbiosis in enhancing the accumulation of phenolics and terpenes has been widely researched as a biotechnological tool to produce phytomass with high concentrations of bioactive compounds with medicinal properties.^2^ Previously, the effect of association with AMF on the production of lectins was established only in roots.^14,15^ Considering the potential of AMF in modulating the lectin profile in other organs, such as the stem, mycorrhizal technology may also be investigated as an alternative to enhance the accumulation of these biomolecules. These proteins are used in biomedicine in many applications, such as drug delivery, producing fluorescent probes, and disease diagnosis.^54^ We verified that to obtain phytomass with elevated content of lectins of biotechnological potential,^53,55^ a cultivation alternative is the use of mycorrhization in the form of a consortium; nevertheless, studies with extracts from mycorrhizal plants associated with isolates must be conducted.

Considering that antimicrobial and anti-inflammatory effects were reported for plant lectins,^56^ a higher production of these with the use of AMF can generate additional benefits to the cultivation of medicinal plants, taking into account that they can act in synergy with other metabolite classes, which have their biosynthesis enhanced by AMF.^57^ Therefore, it is relevant to evaluate the effect of mycorrhizal symbiosis in the production of lectins, considering other plant organs, such as leaves, and hosts from other families, as well as other growth conditions.

## Conclusions

The study’s hypotheses were corroborated, as the association with an AMF consortium can quantitatively and qualitatively modulate lectin accumulation in the host’s stem and that this bioactivity is related to the biosynthesis of some secondary metabolites, with a relationship between enhancing primary and secondary metabolisms, as well as *in vitro* antioxidant activity, and the increase of specific hemagglutinating activity with all tested blood groups. The verified profile of specific hemagglutinating activity of stem extracts, variable depending on blood group, permits suggesting that there is biospecificity in the interaction of lectins and glycans in the cell surfaces promoting the web of agglutinated cells. We still consider that the hemagglutinating effect can result from the action of phenols and carbohydrates synergistically acting or not with lectins. Future work should employ carbohydrate-inhibition assays to confirm lectin specificity and explore AMF–lectin synergies in medicinal plant cultivation.

## Supplementary Material



## Notes

Additional images showing the hemagglutinating activity assays, the hemagglutination pattern caused by the extracts, and a schematization of the experimental design and evaluations (PDF)
