# Supplementary material for: Does Mycorrhizal Biotechnology Modulate Lectin Accumulation in the Stem of Schinus terebinthifolia Raddi Seedlings?
Source: ACS Omega. 2025 Sep 12;10(37):43291–9. doi: 10.1021/acsomega.5c07898 (PMC12461410; doi:10.1021/acsomega.5c07898)
Supplement: Supplementary file 1 [file ao5c07898_si_001.pdf]

Supporting information

Does mycorrhizal biotechnology modulate lectin accumulation in the stem of *Schinus terebinthifolia* Raddi seedlings?

Caio Bezerra Barreto[1], Francisco Chagas Barbalho Neto[2], Carmelo José Albanez Bastos-Filho[3], Qiang-Sheng Wu[4], Michele Dalvina Correia da Silva[5], Fábio Sérgio Barbosa da Silva*[6,1]

[1] Laboratório de Análises, Pesquisas e Estudos em Micorrizas (LAPEM), Programa de Pós-graduação em Biologia Celular e Molecular Aplicada, Universidade de Pernambuco (UPE), Rua Arnóbio Marques, 310, Santo Amaro – Recife, PE, 50100-130, Brazil.

[2] Universidade do Estado do Rio Grande do Norte (UERN), Rua Almino Afonso, 478, Centro – Mossoró, RN, 59610-210, Brazil

[3] Programa de Pós-graduação em Engenharia de Sistemas, Escola Politécnica de Pernambuco, Universidade de Pernambuco (UPE), Rua Benfica, 455, Madalena – Recife, PE, 50720-001, Brazil

[4] Hubei Key Laboratory of Spices and Horticultural Plant Germplasm Innovation and Utilization, College of Horticulture and Gardening, Yangtze University, Jingzhou 434025, China.

[5] Universidade Federal Rural do Semi-Árido (UFERSA), Av. Francisco Mota, 572, Bairro Costa e Silva – Mossoró, RN, 59625-900, Brazil

[6] Universidade de Pernambuco, Av. Agamenon Magalhães, S/N, Santo Amaro – Recife, PE, 50100-010, Brazil.

*E-mail: fabio.barbosa@upe.br

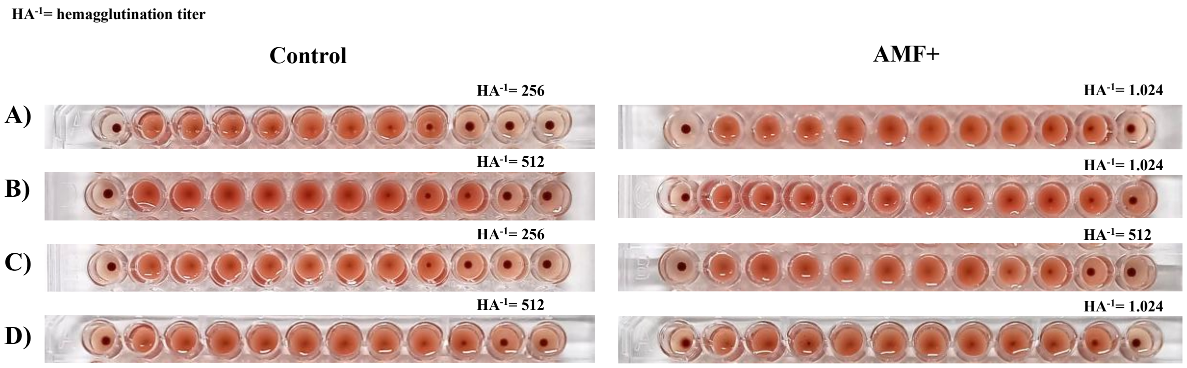

Figure S1. Hemagglutinating activity assays (hemagglutination activity titer, HA^{-1}) of the aqueous stem extract of *Schinus terebinthifolia* Raddi seedlings noninoculated (Control) or inoculated with arbuscular mycorrhizal fungi (AMF+), using glutaraldehyde-fixed human erythrocytes of group A (A), group AB (B), group B (C) and group O (D).

A)

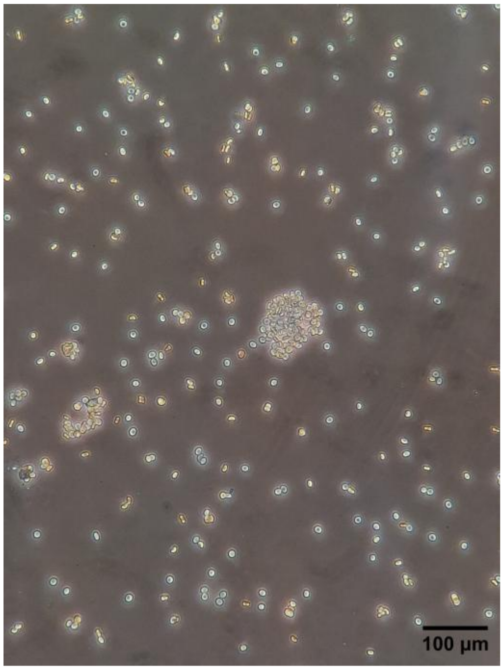

B)

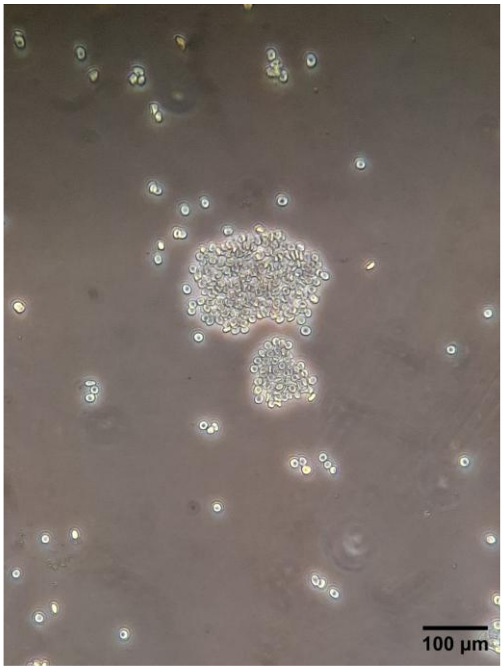

Figure S2. Agglutinating effect of aqueous stem extracts from *Schinus terebinthifolia* Raddi seedlings, noninoculated (Control) or inoculated with arbuscular mycorrhizal fungi (AMF+), on human erythrocytes. A) Hemagglutinating effect of AMF+ plants extract on group AB human erythrocytes. B) Hemagglutinating effect of control plants extract on group O human erythrocytes. Scale bars were added with ImageJ.

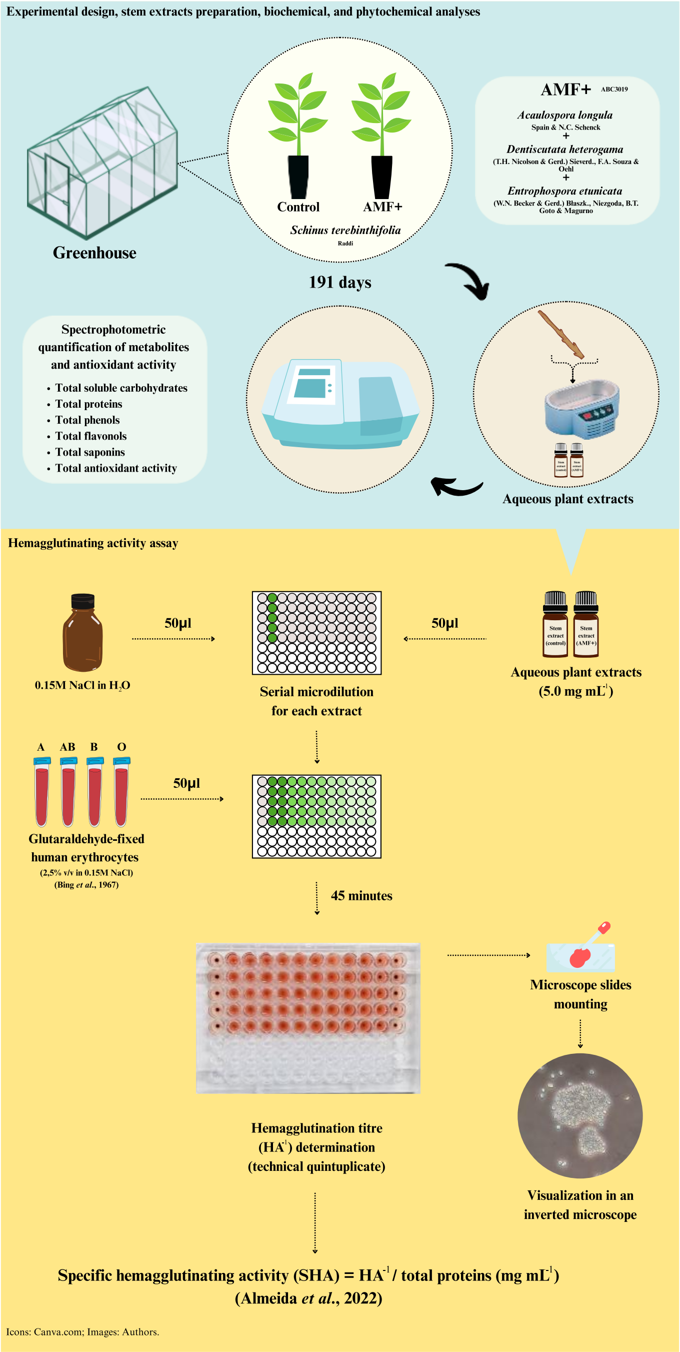

Figure S3. Schematization of the experimental design, preparation of plant extracts, biochemical and phytochemical evaluations, and conduction of hemagglutinating activity assays. AMF= arbuscular mycorrhizal fungi; NaCl= sodium chloride.
